# (Carbazol-9-ido-κ*N*)di­chlorido­(η^5^:η^1^-2,3,4,5-tetra­methyl­penta­fulvene)tantalum(V)

**DOI:** 10.1107/S2414314622012019

**Published:** 2022-12-23

**Authors:** Simon de Graaff, Aylişa Elma, Marc Schmidtmann, Rüdiger Beckhaus

**Affiliations:** a Carl von Ossietzky Universität Oldenburg, Fakultät V - Mathematik und Naturwissenschaften, Institut für Chemie, Carl-von-Ossietzky-Strasse 9-11, D-26111 Oldenburg, Germany; Vienna University of Technology, Austria

**Keywords:** crystal structure, tantalum, penta­fulvene, amide, chloride

## Abstract

The reaction of (η^5^:η^1^-2,3,4,5-tetra­methyl­penta­fulvene)tantalum(V) dicarbazolide chloride (**1**) with etheric HCl results in the formation of the title compound (**2**), [Ta(C_10_H_14_)(C_12_H_8_N)Cl_2_]. The Ta^V^ atom has a distorted tetra­hedral coordination environment in a three-legged piano-stool fashion. The conformation of the penta­fulvene exocyclic C atom to the three other ligands is staggered and not eclipsed, as found in the crystal structure of **1**. Inter­molecular inter­actions include π–π stacking, H⋯π inter­actions and weak C—H⋯Cl hydrogen bonds.

## Abstract 2

In the title compound, the exocyclic penta­fulvene carbon atom coordinates in a staggered conformation relative to the η^1^-ligands.

## Graphical abstract

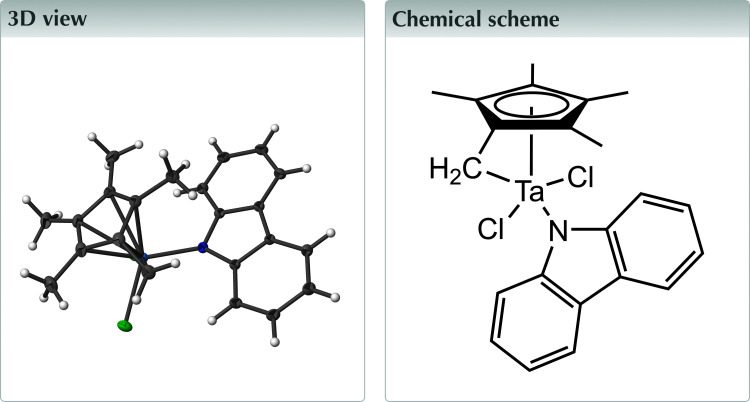

## Structure description

Penta­fulvenes are versatile compounds in organic and organometallic chemistry (Preethalayam *et al.*, 2017 ▸). The latter is dominated by group 4 complexes and their broad scope of consecutive reactions (Beckhaus, 2018 ▸). For group 5 derivatives, a bis­(penta­fulvene)niobium complex was synthesized (Manssen *et al.*, 2018 ▸), and subsequently alkyl­idene (de Graaff *et al.*, 2021 ▸), and ethyl­ene penta­fulvene complexes were investigated (de Graaff *et al.*, 2022 ▸). For tantalum, a series of penta­fulvene complexes has been prepared by C—H activation of a cyclo­penta­dienyl methyl group, also known as ‘tuck-in’ complexes: from deca­methyl tantalocene hydride by oxidative addition of one methyl C—H bond to the metal (Antonelli *et al.*, 1993 ▸) and trapping by elemental sulfur (Brunner *et al.*, 1996 ▸), as well as by rearrangement of a borataalkene tantalocene (Cook *et al.*, 2002 ▸), or Cp*Ta[N(^*i*^Pr)C(NMe_2_)N(^*i*^Pr)](*κ* ^1^-NNMe_2_) (Keane *et al.*, 2013 ▸). Uncommonly, Riley *et al.* (1999 ▸) found the C—H activation at the Cp* ligand of Cp*TaCl_4_ by an amide, synthesizing **1**, η^5^:η^1^-(2,3,4,5-tetra­methyl­penta­fulvene)tantalum(V) dicarbazolide chloride.

**Figure 1:**
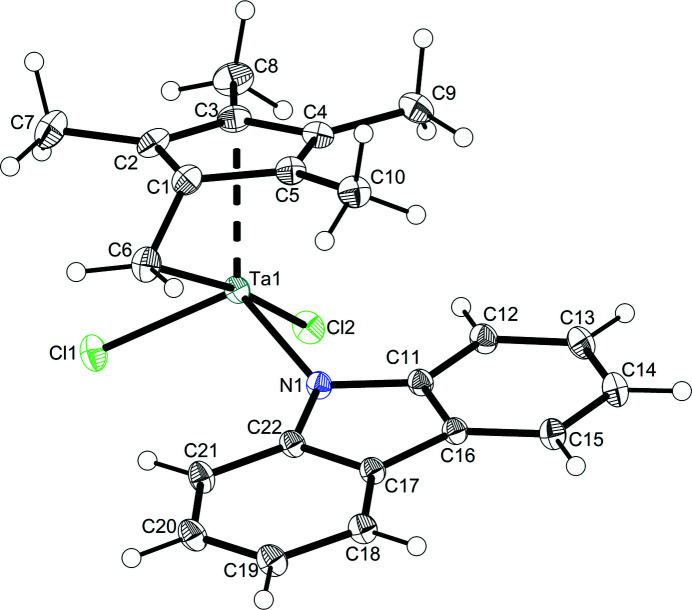
Mol­ecular structure of **2.** Displacement ellipsoids correspond to the 50% probability level.

**Figure 2:**
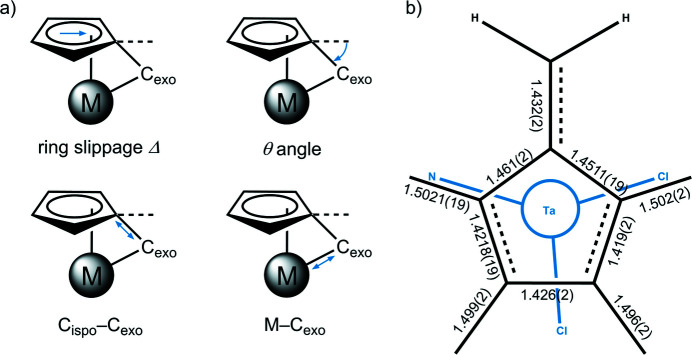
(*a*) Schematic representation of key structural factors characterizing a penta­fulvene complex. (*b*) Schematic drawing of the penta­fulvene ligand above the central tantalum atom. C—C bond lengths of the penta­fulvene ligand are given in Å.

The mol­ecular structure of the title compound **2** is shown in Fig. 1 ▸. The Ta^V^ atom is coordinated in a tetra­hedrally distorted three-legged piano-stool fashion. Two angles between the three η^1^-ligands are smaller [Cl1—Ta1—Cl2: 88.239 (10)°; N1—Ta1—Cl2: 93.54 (3)°], the third being widened due to the direct neighboring of the penta­fulvene exocyclic η^1^-carbon (C6_*exo*_) coordination site [N1—Ta1—Cl1: 114.15 (3)°]. The C6_*exo*_ atom coordinates roughly opposite of Cl2 to the central tantalum atom [C6—Ta1—Cl2: 171.58 (3)°]. Relative to the centroid of the five-membered ring (Ct), the angles to the chloride ligands are smaller than to the nitro­gen ligands [Cl1—Ta1—Ct: 116.715 (8)°; Cl2—Ta1—Ct: 115.508 (9)°; N1—Ta1—Ct: 121.012 (3)°]. The bond length Ta1—N1 [2.0433 (9) Å] and the sum of angles at N1 [347.1 (2)°] indicates a weak inter­action of the nitro­gen lone pair with the metal. The penta­fulvene coordinates in a π-η^5^:σ-η^1^ fashion and exhibits typical distortion parameters (Fig. 2 ▸ *a*). The C—C bond lengths within the penta­fulvene are summarized in Fig. 2 ▸ *b*. The penta­fulvene has a ring slippage Δ of 0.31 Å and a θ angle of the C_*ipso*_—C_*exo*_ bond out of the plane of the five-membered ring of 36.30 (12)°. The C_*ipso*_—C_*exo*_ bond is a single to double bond [C1–C6: 1.4311 (7) Å; Allen *et al.*, 1987 ▸] and the distance between the central tantalum atom and the C_*exo*_ atom exceeds the sum of their covalent radii [Ta1—C6: 2.379 (11) Å; sum of covalent radii 2.11 Å (Pyykkö & Atsumi, 2009 ▸)].

**Figure 3:**
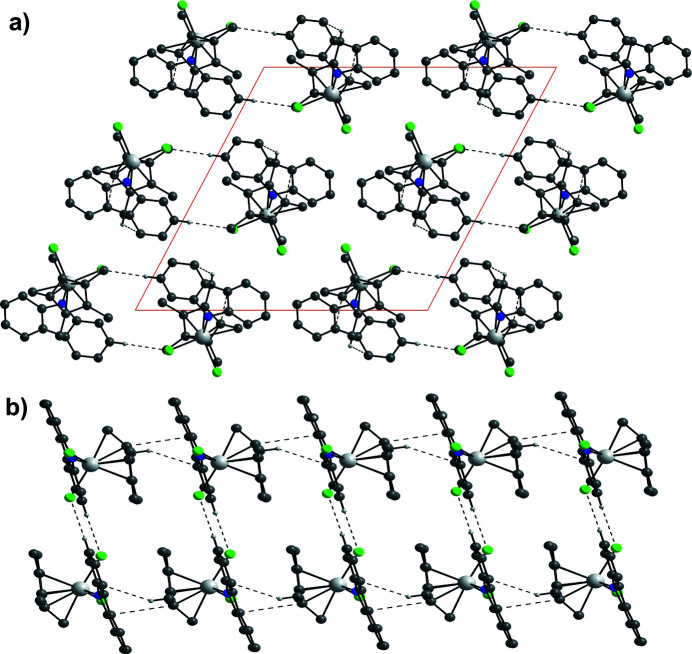
(*a*) A view along the *b* axis showing the packing of mol­ecule pairs of **2** inter­acting *via* C—H⋯Cl hydrogen bonds. (*b*) Double chains of **2** formed by π–π stacking and H⋯π inter­actions. Color code: C dark gray, H white, Cl green, N blue, Ta light gray.

On the supra­molecular level, around an inversion center, two mol­ecules mutually inter­act *via* two weak carbazolide C—H⋯Cl hydrogen bonds [H13⋯Cl2: 2.7719 (12) Å; Fig. 3 ▸ *a*]. Consequently, the Ta1—Cl2 bond [2.3965 (3) Å) is longer than the Ta1—Cl1 bond [2.3452 (3) Å]. These pairs form a double-chain (Fig. 3 ▸ *b*), linked by supra­molecular contacts of the penta­fulvene and the carbazolide ligands *via* π–π stacking [C1⋯C17: 3.3867 (15) Å] and an H⋯π inter­action [C15⋯H10*c*: 2.773 (6) Å]. Numerical details of other hydrogen-bonding inter­actions are summerized in Table 1 ▸.

## Synthesis and crystallization

All steps were carried out under a dry argon atmosphere in a glovebox and under a dry nitro­gen atmosphere using Schlenk techniques. Compound **1** was prepared according to Riley *et al.* (1999 ▸), substituting potassium for lithium. Solvents were dried according to standard procedures over Na/K alloy with benzo­phenone as indicator and distilled under a nitro­gen atmosphere. Etheric HCl was acquired from Sigma-Aldrich.

Complex **1** (550 mg, 0.8 mmol) was dissolved in tetra­hydro­furan (20 ml) and cooled to 223 K. One equivalent of etheric HCl (2 *M*, 0.4 ml, 0.8 mmol) was added dropwise and the solution was slowly brought to room temperature. After stirring over night, the solvents were removed *in vacuo* and the residue was extracted with toluene (10 ml). The solution was diluted with *n*-hexane (10 ml) and stored at 277 K for three days to yield a red crystalline material containing **1** and **2** (1:1). ^1^H NMR (300 MHz, C_6_D_6_, 294 K): δ = 0.79 (*s*, 3H, **1**), 0.85 (*s*, 3H, **1**), 1.27 (*s*, 6H, **2**), 1.49 (*s*, 3H, **1**), 1.53 (*s*, 6H, **2**), 2.13 (*s*, 3H, **1**), 2.62 (*s*, 2H, **2**), 3.27 (*d*, ^2^ *J*_HH_ = 7.4 Hz, 1H, **1**), 3.65 (*d*, ^2^ *J*_HH_ = 7.4 Hz, 1H, **1**), 6.30–8.29 (aromatic signals unassigned) p.p.m.

## Refinement

Crystal data, data collection and structure refinement details are summarized in Table 2 ▸. Refinement using *SHELXL* (Sheldrick, 2015 ▸) and anisotropic displacement parameters results in high residual electron densities next to the tantalum atom (maximum: 4.16 e^−^ Å^−3^; minimum: −2.83 e^−^ Å^−3^). Refinement with *OLEX2* (Bourhis *et al.*, 2015 ▸) provides the possibility to refine the tantalum atom with anharmonic displacement parameters. Thereby, the residue electron density is lowered significantly (maximum: 1.28 e^−^ Å^−3^; minimum: −1.24 e^−^ Å^−3^). Refining all atoms anharmonically was dismissed, because it lowers the reliability factors only marginally, but more than triples the refinement parameters (263 *versus* 888 parameters).

## Supplementary Material

Crystal structure: contains datablock(s) I. DOI: 10.1107/S2414314622012019/wm4176sup1.cif

Structure factors: contains datablock(s) I. DOI: 10.1107/S2414314622012019/wm4176Isup2.hkl

CCDC reference: 2231842

Additional supporting information:  crystallographic information; 3D view; checkCIF report

## Figures and Tables

**Table 1:** Hydrogen-bond geometry (Å, °)

*D*—H⋯*A*	*D*—H	H⋯*A*	*D*⋯*A*	*D*—H⋯*A*
C8—H8*b*⋯Cl2^i^	0.98	2.91 (1)	3.7119 (14)	140 (1)
C12—H12⋯Cl2	0.95	2.64 (1)	3.3663 (12)	134 (1)
C13—H13⋯Cl2^ii^	0.95	2.77 (1)	3.7217 (12)	179 (1)
C15—H15⋯Cl2^iii^	0.95	2.86 (1)	3.7923 (12)	167 (1)
C18—H18⋯Cl1^iii^	0.95	3.13 (1)	3.7645 (11)	126 (1)
C18—H18⋯Cl2^iii^	0.95	2.85 (1)	3.7795 (12)	167 (1)
C19—H19⋯Cl1^iii^	0.95	3.08 (1)	3.7479 (12)	128 (1)
C20—H20⋯Cl1^iv^	0.95	2.95 (1)	3.5548 (12)	123 (1)

Symmetry codes: (i) 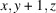 ; (ii) 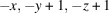 ; (iii) 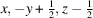 ; (iv) 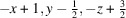 .

**Table 2:** Experimental details

Crystal data	
Chemical formula	[Ta(C_10_H_14_)(C_12_H_8_N)Cl_2_]
*M*_r_	552.28
Crystal system, space group	Monoclinic, *P*2_1_/*c*
Temperature (K)	100
*a*, *b*, *c* (Å)	17.8422 (12), 7.3442 (5), 16.7885 (11)
β (°)	117.950 (2)
*V* (Å^3^)	1943.3 (2)
*Z*	4
Radiation type	Mo *K*α
μ (mm^−1^)	5.94
Crystal size (mm)	0.12 × 0.11 × 0.05

Data collection	
Diffractometer	Bruker Photon III CPAD
Absorption correction	Multi-scan (*SADABS*; Krause *et al.*, 2015 ▸)
*T*_min_, *T*_max_	0.511, 0.651
No. of measured, independent and observed [*I* ≥ 2u(*I*)] reflections	128718, 12240, 11363
*R*_int_	0.043
(sin θ/λ)_max_ (Å^−1^)	0.909

Refinement	
*R*[*F* ^2^ > 2σ(*F* ^2^)], *wR*(*F* ^2^), *S*	0.016, 0.044, 1.11
No. of reflections	12240
No. of parameters	264
H-atom treatment	H-atom parameters constrained
Δρ_max_, Δρ_min_ (e Å^−3^)	1.28, −1.24

Computer programs: *APEX3* and *SAINT* (Bruker, 2016 ▸), *SHELXS* (Sheldrick, 2008 ▸), and *OLEX2* (Bourhis *et al.*, 2015 ▸).

## full crystallographic data

### Crystal data

**Table:**

[Ta(C_10_H_14_)(C_12_H_8_N)Cl_2_]	*F*(000) = 1072.380
*M**_r_* = 552.28	*D*_x_ = 1.888 Mg m^−^^3^
Monoclinic, *P*2_1_/*c*	Mo *K*α radiation, λ = 0.71073 Å
*a* = 17.8422 (12) Å	Cell parameters from 9727 reflections
*b* = 7.3442 (5) Å	θ = 2.6–40.3°
*c* = 16.7885 (11) Å	µ = 5.94 mm^−^^1^
β = 117.950 (2)°	*T* = 100 K
*V* = 1943.3 (2) Å^3^	Block, red
*Z* = 4	0.12 × 0.11 × 0.05 mm

### Data collection

**Table:**

Bruker Photon III CPAD diffractometer	11363 reflections with *I*≥ 2θ(*I*)
φ and ω scans	*R*_int_ = 0.043
Absorption correction: multi-scan (*SADABS*; Krause *et al.*, 2015)	θ_max_ = 40.3°, θ_min_ = 2.4°
*T*_min_ = 0.511, *T*_max_ = 0.651	*h* = −32→32
128718 measured reflections	*k* = −13→13
12240 independent reflections	*l* = −30→30

### Refinement

**Table:**

Refinement on *F*^2^	35 constraints
Least-squares matrix: full	Primary atom site location: structure-invariant direct methods
*R*[*F*^2^ > 2σ(*F*^2^)] = 0.016	H-atom parameters constrained
*wR*(*F*^2^) = 0.044	*w* = 1/[σ^2^(*F*_o_^2^) + (0.0164*P*)^2^ + 1.1947*P*] where *P* = (*F*_o_^2^ + 2*F*_c_^2^)/3
*S* = 1.11	(Δ/σ)_max_ = −0.001
12240 reflections	Δρ_max_ = 1.28 e Å^−^^3^
264 parameters	Δρ_min_ = −1.24 e Å^−^^3^
0 restraints	

### Fractional atomic coordinates and isotropic or equivalent isotropic displacement parameters (Å^2^)

**Table:**

	*x*	*y*	*z*	*U*_iso_*/*U*_eq_	
Ta1	0.278227 (6)	0.666931 (14)	0.609660 (6)	0.01317 (5)	
Cl1	0.392598 (18)	0.63539 (4)	0.754919 (18)	0.02060 (5)	
Cl2	0.188124 (17)	0.54264 (4)	0.666091 (19)	0.01858 (5)	
N1	0.26155 (5)	0.45000 (13)	0.52677 (6)	0.01386 (12)	
C1	0.31669 (7)	0.90414 (15)	0.55660 (7)	0.01712 (16)	
C2	0.30629 (8)	0.98409 (15)	0.62994 (8)	0.01875 (17)	
C3	0.21917 (8)	0.97380 (15)	0.60722 (8)	0.01822 (17)	
C4	0.17348 (7)	0.89795 (15)	0.51906 (8)	0.01707 (16)	
C5	0.23188 (7)	0.85726 (15)	0.48611 (7)	0.01642 (16)	
C6	0.38444 (7)	0.77942 (18)	0.57472 (8)	0.01934 (18)	
H6a	0.44053 (7)	0.80823 (18)	0.62603 (8)	0.0232 (2)*	
H6b	0.38711 (7)	0.72515 (18)	0.52213 (8)	0.0232 (2)*	
C7	0.37542 (10)	1.06857 (19)	0.71354 (9)	0.0256 (2)	
H7a	0.3710 (6)	1.20151 (19)	0.7085 (4)	0.0384 (3)*	
H7b	0.3696 (6)	1.0293 (16)	0.76616 (15)	0.0384 (3)*	
H7c	0.43078 (10)	1.0301 (16)	0.7205 (5)	0.0384 (3)*	
C8	0.17969 (10)	1.03756 (19)	0.66363 (10)	0.0256 (2)	
H8a	0.22378 (17)	1.052 (2)	0.7263 (2)	0.0384 (3)*	
H8b	0.1516 (9)	1.1549 (10)	0.6408 (7)	0.0384 (3)*	
H8c	0.1378 (7)	0.9479 (10)	0.6608 (8)	0.0384 (3)*	
C9	0.07868 (8)	0.88368 (19)	0.46864 (9)	0.0225 (2)	
H9a	0.06216 (9)	0.8053 (15)	0.4158 (5)	0.0337 (3)*	
H9b	0.05769 (11)	0.8313 (17)	0.5081 (3)	0.0337 (3)*	
H9c	0.05421 (9)	1.0052 (3)	0.4490 (8)	0.0337 (3)*	
C10	0.21185 (8)	0.78707 (18)	0.39424 (7)	0.01993 (18)	
H10a	0.2608 (3)	0.7197 (15)	0.39786 (18)	0.0299 (3)*	
H10b	0.1626 (5)	0.7060 (14)	0.3724 (4)	0.0299 (3)*	
H10c	0.1992 (8)	0.8897 (2)	0.3526 (2)	0.0299 (3)*	
C11	0.18132 (6)	0.39330 (14)	0.45672 (7)	0.01415 (14)	
C12	0.10037 (7)	0.42830 (16)	0.44654 (8)	0.01689 (16)	
H12	0.09314 (7)	0.49491 (16)	0.49097 (8)	0.02027 (19)*	
C13	0.03042 (7)	0.36314 (17)	0.36962 (8)	0.01928 (18)	
H13	−0.02505 (7)	0.38633 (17)	0.36168 (8)	0.0231 (2)*	
C14	0.04027 (7)	0.26427 (18)	0.30388 (8)	0.01979 (18)	
H14	−0.00838 (7)	0.22206 (18)	0.25180 (8)	0.0238 (2)*	
C15	0.12064 (7)	0.22749 (16)	0.31426 (7)	0.01721 (16)	
H15	0.12740 (7)	0.16001 (16)	0.26977 (7)	0.02065 (19)*	
C16	0.19144 (6)	0.29109 (14)	0.39104 (7)	0.01395 (14)	
C17	0.28193 (6)	0.27368 (14)	0.42311 (7)	0.01377 (14)	
C18	0.32964 (7)	0.18151 (15)	0.38960 (8)	0.01630 (16)	
H18	0.30272 (7)	0.12006 (15)	0.33329 (8)	0.01957 (19)*	
C19	0.41763 (7)	0.18178 (16)	0.44069 (8)	0.01833 (17)	
H19	0.45125 (7)	0.12051 (16)	0.41887 (8)	0.0220 (2)*	
C20	0.45710 (7)	0.27159 (17)	0.52402 (8)	0.01864 (17)	
H20	0.51720 (7)	0.26906 (17)	0.55802 (8)	0.0224 (2)*	
C21	0.41031 (7)	0.36438 (16)	0.55811 (7)	0.01696 (16)	
H21	0.43742 (7)	0.42398 (16)	0.61491 (7)	0.02035 (19)*	
C22	0.32212 (6)	0.36687 (14)	0.50595 (7)	0.01367 (14)	

### Atomic displacement parameters (Å^2^)

**Table:**

	*U*^11^	*U*^22^	*U*^33^	*U*^12^	*U*^13^	*U*^23^
Ta1	0.01119 (6)	0.01644 (8)	0.01408 (7)	0.00048 (3)	0.00775 (5)	0.00024 (3)
Cl1	0.01654 (10)	0.02804 (12)	0.01445 (9)	−0.00206 (9)	0.00495 (8)	0.00104 (8)
Cl2	0.01698 (10)	0.02367 (11)	0.01952 (10)	0.00074 (8)	0.01225 (8)	0.00389 (8)
N1	0.0117 (3)	0.0159 (3)	0.0152 (3)	0.0003 (2)	0.0074 (2)	−0.0015 (2)
C1	0.0197 (4)	0.0167 (4)	0.0181 (4)	−0.0030 (3)	0.0115 (3)	0.0003 (3)
C2	0.0256 (5)	0.0139 (4)	0.0200 (4)	−0.0037 (3)	0.0135 (4)	−0.0021 (3)
C3	0.0228 (4)	0.0155 (4)	0.0200 (4)	0.0021 (3)	0.0130 (4)	−0.0002 (3)
C4	0.0187 (4)	0.0159 (4)	0.0182 (4)	0.0030 (3)	0.0100 (3)	0.0023 (3)
C5	0.0192 (4)	0.0166 (4)	0.0155 (4)	0.0004 (3)	0.0098 (3)	0.0016 (3)
C6	0.0165 (4)	0.0244 (5)	0.0207 (4)	−0.0033 (3)	0.0118 (3)	−0.0016 (4)
C7	0.0300 (6)	0.0238 (5)	0.0232 (5)	−0.0088 (4)	0.0127 (4)	−0.0072 (4)
C8	0.0324 (6)	0.0230 (5)	0.0292 (6)	0.0029 (4)	0.0210 (5)	−0.0041 (4)
C9	0.0192 (4)	0.0235 (5)	0.0250 (5)	0.0062 (4)	0.0106 (4)	0.0036 (4)
C10	0.0243 (5)	0.0215 (4)	0.0153 (4)	0.0007 (4)	0.0105 (4)	0.0018 (3)
C11	0.0122 (3)	0.0154 (4)	0.0151 (3)	0.0006 (3)	0.0066 (3)	−0.0001 (3)
C12	0.0128 (3)	0.0197 (4)	0.0194 (4)	0.0011 (3)	0.0086 (3)	−0.0007 (3)
C13	0.0121 (4)	0.0240 (5)	0.0207 (4)	0.0016 (3)	0.0068 (3)	0.0011 (4)
C14	0.0135 (4)	0.0251 (5)	0.0180 (4)	−0.0009 (3)	0.0050 (3)	−0.0003 (4)
C15	0.0153 (4)	0.0204 (4)	0.0146 (4)	−0.0005 (3)	0.0060 (3)	−0.0010 (3)
C16	0.0126 (3)	0.0154 (3)	0.0144 (3)	0.0004 (3)	0.0068 (3)	−0.0001 (3)
C17	0.0124 (3)	0.0156 (4)	0.0142 (3)	0.0009 (3)	0.0069 (3)	−0.0001 (3)
C18	0.0154 (4)	0.0195 (4)	0.0162 (4)	0.0014 (3)	0.0093 (3)	−0.0011 (3)
C19	0.0154 (4)	0.0232 (5)	0.0194 (4)	0.0031 (3)	0.0106 (3)	0.0000 (3)
C20	0.0128 (4)	0.0240 (5)	0.0195 (4)	0.0034 (3)	0.0079 (3)	0.0006 (3)
C21	0.0121 (3)	0.0228 (4)	0.0155 (4)	0.0013 (3)	0.0061 (3)	−0.0007 (3)
C22	0.0118 (3)	0.0162 (4)	0.0138 (3)	0.0012 (3)	0.0068 (3)	−0.0001 (3)

### Geometric parameters (Å, º)

**Table:**

Ta1—Cl1	2.3452 (3)	C8—H8c	0.9800
Ta1—Cl2	2.3965 (3)	C9—H9a	0.9800
Ta1—N1	2.0433 (9)	C9—H9b	0.9800
Ta1—C1	2.2074 (11)	C9—H9c	0.9800
Ta1—C2	2.3732 (11)	C10—H10a	0.9800
Ta1—C3	2.4801 (11)	C10—H10b	0.9800
Ta1—C4	2.4536 (11)	C10—H10c	0.9800
Ta1—C5	2.3091 (11)	C11—C12	1.3965 (14)
Ta1—C6	2.3791 (11)	C11—C16	1.4137 (14)
N1—C11	1.4238 (13)	C12—H12	0.9500
N1—C22	1.4202 (13)	C12—C13	1.3941 (16)
C1—C2	1.4525 (16)	C13—H13	0.9500
C1—C5	1.4594 (16)	C13—C14	1.3994 (18)
C1—C6	1.4311 (17)	C14—H14	0.9500
C2—C3	1.4183 (18)	C14—C15	1.3876 (16)
C2—C7	1.5012 (18)	C15—H15	0.9500
C3—C4	1.4263 (16)	C15—C16	1.3960 (15)
C3—C8	1.4959 (17)	C16—C17	1.4486 (14)
C4—C5	1.4217 (16)	C17—C18	1.3957 (15)
C4—C9	1.4988 (17)	C17—C22	1.4079 (14)
C5—C10	1.5020 (16)	C18—H18	0.9500
C6—H6a	0.9900	C18—C19	1.3922 (16)
C6—H6b	0.9900	C19—H19	0.9500
C7—H7a	0.9800	C19—C20	1.4015 (17)
C7—H7b	0.9800	C20—H20	0.9500
C7—H7c	0.9800	C20—C21	1.3917 (16)
C8—H8a	0.9800	C21—H21	0.9500
C8—H8b	0.9800	C21—C22	1.3969 (15)
			
Cl2—Ta1—Cl1	88.239 (10)	C10—C5—C4	127.35 (11)
N1—Ta1—Cl1	114.15 (3)	C1—C6—Ta1	65.39 (6)
N1—Ta1—Cl2	93.54 (3)	H6a—C6—Ta1	117.19 (3)
C1—Ta1—Cl1	102.36 (3)	H6a—C6—C1	117.19 (7)
C1—Ta1—Cl2	148.56 (3)	H6b—C6—Ta1	117.19 (3)
C1—Ta1—N1	108.30 (4)	H6b—C6—C1	117.19 (6)
C2—Ta1—Cl1	85.73 (3)	H6b—C6—H6a	114.2
C2—Ta1—Cl2	116.91 (3)	H7a—C7—C2	109.5
C2—Ta1—N1	144.74 (4)	H7b—C7—C2	109.5
C2—Ta1—C1	36.75 (4)	H7b—C7—H7a	109.5
C3—Ta1—Cl1	105.24 (3)	H7c—C7—C2	109.5
C3—Ta1—Cl2	89.66 (3)	H7c—C7—H7a	109.5
C3—Ta1—N1	140.55 (4)	H7c—C7—H7b	109.5
C3—Ta1—C1	59.08 (4)	H8a—C8—C3	109.5
C3—Ta1—C2	33.89 (4)	H8b—C8—C3	109.5
C4—Ta1—Cl1	138.73 (3)	H8b—C8—H8a	109.5
C4—Ta1—Cl2	92.94 (3)	H8c—C8—C3	109.5
C4—Ta1—N1	106.95 (4)	H8c—C8—H8a	109.5
C4—Ta1—C1	59.64 (4)	H8c—C8—H8b	109.5
C4—Ta1—C2	57.21 (4)	H9a—C9—C4	109.5
C4—Ta1—C3	33.60 (4)	H9b—C9—C4	109.5
C5—Ta1—Cl1	139.87 (3)	H9b—C9—H9a	109.5
C5—Ta1—Cl2	124.16 (3)	H9c—C9—C4	109.5
C5—Ta1—N1	89.07 (4)	H9c—C9—H9a	109.5
C5—Ta1—C1	37.62 (4)	H9c—C9—H9b	109.5
C5—Ta1—C2	59.83 (4)	H10a—C10—C5	109.5
C5—Ta1—C3	57.63 (4)	H10b—C10—C5	109.5
C5—Ta1—C4	34.57 (4)	H10b—C10—H10a	109.5
C6—Ta1—Cl1	83.41 (3)	H10c—C10—C5	109.5
C6—Ta1—Cl2	171.58 (3)	H10c—C10—H10a	109.5
C6—Ta1—N1	88.93 (4)	H10c—C10—H10b	109.5
C6—Ta1—C1	36.12 (4)	C12—C11—N1	128.98 (9)
C6—Ta1—C2	63.62 (4)	C16—C11—N1	110.67 (8)
C6—Ta1—C3	93.54 (4)	C16—C11—C12	120.34 (9)
C6—Ta1—C4	94.03 (4)	H12—C12—C11	120.80 (6)
C6—Ta1—C5	63.87 (4)	C13—C12—C11	118.41 (10)
C11—N1—Ta1	124.03 (7)	C13—C12—H12	120.80 (7)
C22—N1—Ta1	128.17 (7)	H13—C13—C12	119.34 (7)
C22—N1—C11	104.92 (8)	C14—C13—C12	121.33 (10)
C2—C1—Ta1	77.85 (6)	C14—C13—H13	119.34 (6)
C5—C1—Ta1	74.97 (6)	H14—C14—C13	119.79 (6)
C5—C1—C2	106.66 (10)	C15—C14—C13	120.42 (10)
C6—C1—Ta1	78.49 (7)	C15—C14—H14	119.79 (7)
C6—C1—C2	120.62 (10)	H15—C15—C14	120.48 (7)
C6—C1—C5	118.22 (10)	C16—C15—C14	119.04 (10)
C1—C2—Ta1	65.41 (6)	C16—C15—H15	120.48 (6)
C3—C2—Ta1	77.19 (6)	C15—C16—C11	120.45 (9)
C3—C2—C1	108.05 (10)	C17—C16—C11	106.53 (8)
C7—C2—Ta1	124.43 (9)	C17—C16—C15	133.01 (10)
C7—C2—C1	125.74 (12)	C18—C17—C16	132.62 (9)
C7—C2—C3	126.17 (11)	C22—C17—C16	106.69 (8)
C2—C3—Ta1	68.92 (6)	C22—C17—C18	120.63 (9)
C4—C3—Ta1	72.18 (6)	H18—C18—C17	120.80 (6)
C4—C3—C2	108.73 (10)	C19—C18—C17	118.40 (10)
C8—C3—Ta1	126.62 (8)	C19—C18—H18	120.80 (6)
C8—C3—C2	126.51 (11)	H19—C19—C18	119.68 (6)
C8—C3—C4	124.72 (11)	C20—C19—C18	120.63 (10)
C3—C4—Ta1	74.22 (6)	C20—C19—H19	119.68 (6)
C5—C4—Ta1	67.15 (6)	H20—C20—C19	119.22 (6)
C5—C4—C3	108.62 (10)	C21—C20—C19	121.56 (10)
C9—C4—Ta1	129.19 (8)	C21—C20—H20	119.22 (6)
C9—C4—C3	123.93 (10)	H21—C21—C20	121.14 (6)
C9—C4—C5	127.19 (11)	C22—C21—C20	117.72 (10)
C1—C5—Ta1	67.41 (6)	C22—C21—H21	121.14 (6)
C4—C5—Ta1	78.28 (6)	C17—C22—N1	110.98 (8)
C4—C5—C1	107.78 (10)	C21—C22—N1	127.94 (9)
C10—C5—Ta1	121.82 (8)	C21—C22—C17	121.02 (9)
C10—C5—C1	124.80 (10)		
			
Ta1—N1—C11—C12	−21.34 (10)	N1—C11—C16—C15	−177.53 (9)
Ta1—N1—C11—C16	157.54 (8)	N1—C11—C16—C17	3.18 (9)
Ta1—N1—C22—C17	−156.81 (9)	N1—C22—C17—C16	−2.47 (10)
Ta1—N1—C22—C21	25.74 (11)	N1—C22—C17—C18	−179.87 (9)
Ta1—C1—C2—C3	−66.06 (7)	N1—C22—C21—C20	179.22 (12)
Ta1—C1—C2—C7	115.89 (8)	C1—C2—C3—C4	−3.05 (10)
Ta1—C1—C5—C4	68.63 (7)	C1—C2—C3—C8	179.13 (9)
Ta1—C1—C5—C10	−113.97 (7)	C1—C5—C4—C3	1.63 (10)
Ta1—C2—C1—C5	70.04 (7)	C1—C5—C4—C9	175.84 (9)
Ta1—C2—C1—C6	−68.61 (7)	C2—C3—C4—C5	0.87 (10)
Ta1—C2—C3—C4	−61.51 (7)	C2—C3—C4—C9	−173.56 (9)
Ta1—C2—C3—C8	120.67 (8)	C3—C4—C5—C10	−175.68 (9)
Ta1—C3—C2—C1	58.46 (7)	C11—C12—C13—C14	0.24 (13)
Ta1—C3—C2—C7	−123.51 (8)	C11—C16—C15—C14	−0.73 (13)
Ta1—C3—C4—C5	−58.60 (7)	C11—C16—C17—C18	176.53 (8)
Ta1—C3—C4—C9	126.96 (7)	C11—C16—C17—C22	−0.43 (10)
Ta1—C4—C3—C2	59.48 (7)	C12—C13—C14—C15	0.48 (15)
Ta1—C4—C3—C8	−122.65 (8)	C13—C14—C15—C16	−0.23 (14)
Ta1—C4—C5—C1	−61.41 (7)	C14—C15—C16—C17	178.34 (10)
Ta1—C4—C5—C10	121.28 (7)	C15—C16—C17—C18	−2.63 (16)
Ta1—C5—C1—C2	−72.07 (7)	C15—C16—C17—C22	−179.59 (14)
Ta1—C5—C1—C6	67.75 (7)	C16—C17—C18—C19	−175.66 (13)
Ta1—C5—C4—C3	63.04 (7)	C16—C17—C22—C21	175.18 (9)
Ta1—C5—C4—C9	−122.75 (7)	C17—C18—C19—C20	0.42 (13)
Ta1—C6—C1—C2	68.27 (7)	C17—C22—C21—C20	2.00 (12)
Ta1—C6—C1—C5	−65.82 (7)	C18—C19—C20—C21	−0.59 (14)
N1—C11—C12—C13	177.59 (12)	C19—C20—C21—C22	−0.62 (14)

### Hydrogen-bond geometry (Å, º)

**Table:**

*D*—H···*A*	*D*—H	H···*A*	*D*···*A*	*D*—H···*A*
C8—H8*b*···Cl2^i^	0.98	2.91 (1)	3.7119 (14)	140 (1)
C12—H12···Cl2	0.95	2.64 (1)	3.3663 (12)	134 (1)
C13—H13···Cl2^ii^	0.95	2.77 (1)	3.7217 (12)	179 (1)
C15—H15···Cl2^iii^	0.95	2.86 (1)	3.7923 (12)	167 (1)
C18—H18···Cl1^iii^	0.95	3.13 (1)	3.7645 (11)	126 (1)
C18—H18···Cl2^iii^	0.95	2.85 (1)	3.7795 (12)	167 (1)
C19—H19···Cl1^iii^	0.95	3.08 (1)	3.7479 (12)	128 (1)
C20—H20···Cl1^iv^	0.95	2.95 (1)	3.5548 (12)	123 (1)

Symmetry codes: (i) *x*, *y*+1, *z*; (ii) −*x*, −*y*+1, −*z*+1; (iii) *x*, −*y*+1/2, *z*−1/2; (iv) −*x*+1, *y*−1/2, −*z*+3/2.
